# APC/C ^Cdh1p^ and Slx5p/Slx8p ubiquitin ligases confer resistance to aminoglycoside hygromycin B in *Saccharomyces cerevisiae*

**DOI:** 10.17912/micropub.biology.000547

**Published:** 2022-03-24

**Authors:** Ellen M. Doss, Mary E. Tragesser-Tiña, Yanru Huang, Philip J. Smaldino, Jason D. True, Ashley L. Kalinski, Eric M. Rubenstein

**Affiliations:** 1 Ball State University, Department of Biology

## Abstract

Multiple ubiquitin ligases with nuclear substrates promote regulated protein degradation and turnover of protein quality control (PQC) substrates. We hypothesized that two ubiquitin ligases with nuclear substrates – the anaphase-promoting complex/cyclosome with the Cdh1p substrate recognition factor (APC/C ^Cdh1p^ ) and the Slx5p/Slx8p SUMO-targeted ubiquitin ligase – contribute to PQC. We predicted yeast lacking subunits of these enzymes would exhibit compromised growth in the presence of hygromycin B, which reduces translational fidelity. We observed that loss of Cdh1p, Slx5p, or Slx8p sensitizes yeast to hygromycin B to a similar extent as loss of two ubiquitin ligases with characterized roles in nuclear PQC and hygromycin B resistance. In addition to their well-characterized function in regulated protein degradation, our results are consistent with prominent roles for both APC/C ^Cdh1p^ and Slx5p/Slx8p in PQC.

**Figure 1. CDH1 , SLX5 , and SLX8 confer resistance to hygromycin B:**
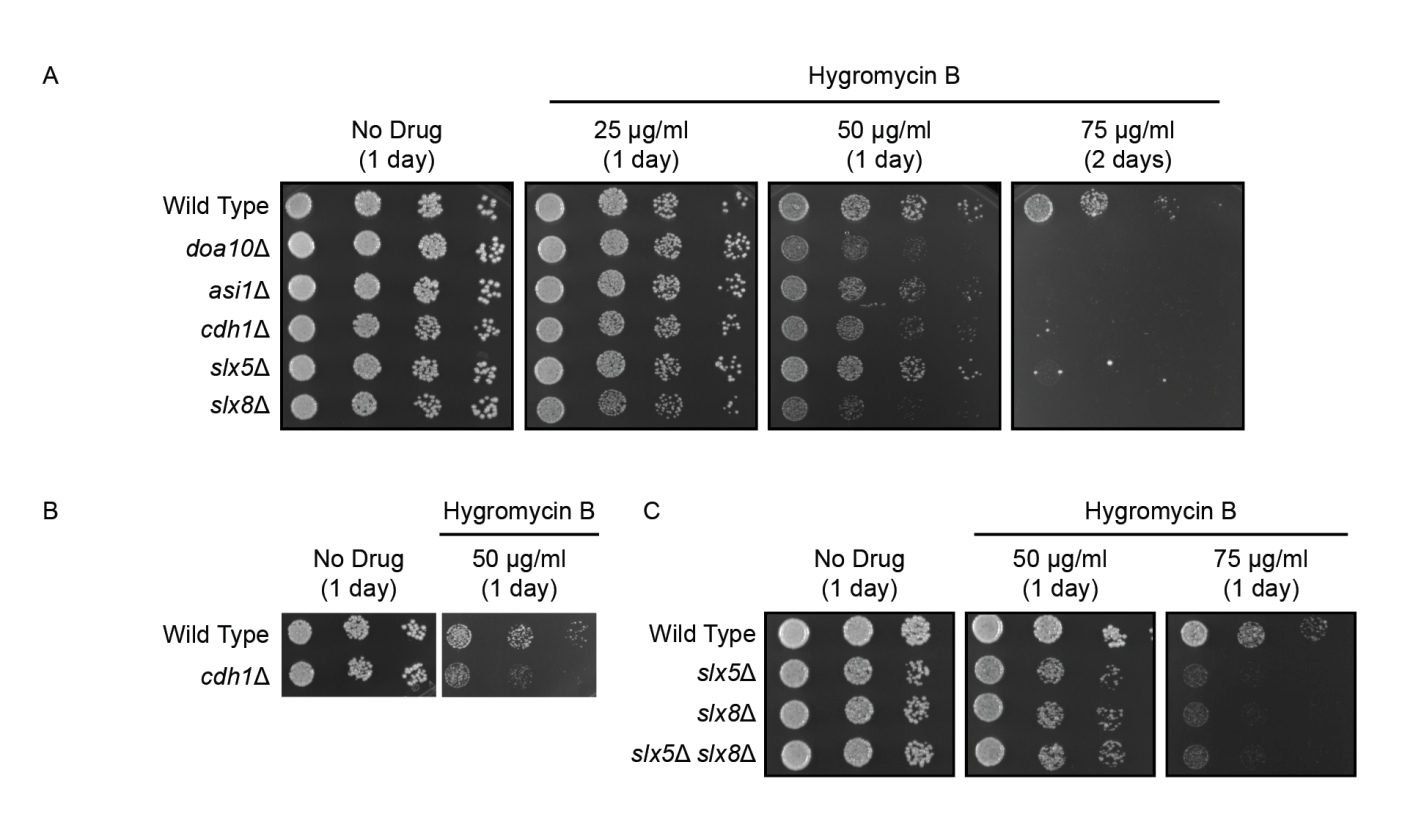
**(A-C)** Sixfold serial dilutions of yeast with indicated genotypes were spotted onto agar plates containing rich growth medium lacking (No Drug) or supplemented with indicated concentrations of hygromycin B. Plates were incubated at 30°C and imaged after 1-2 days. Experiments were performed in triplicate.

## Description

The ubiquitin-proteasome system (UPS) mediates both regulatory and quality control protein degradation (Finley et al., 2012; Kleiger and Mayor, 2014). In the UPS, ubiquitin ligase enzymes covalently attach multiple copies of the small protein ubiquitin to substrate proteins. Polyubiquitylated proteins are degraded by the 26S proteasome.

Several ubiquitin ligases contribute to the degradation of nuclear proteins in *Saccharomyces cerevisiae* (Breckel and Hochstrasser, 2021). The integral membrane Doa10p ubiquitin ligase resides in the inner nuclear and endoplasmic reticulum (ER) membranes and targets both nuclear and ER proteins for degradation (Ravid et al., 2006; Swanson et al., 2001). Doa10p substrates include naturally short-lived proteins and misfolded polypeptides (Huyer et al., 2004; Swanson et al., 2001). The heterotrimeric transmembrane Asi ubiquitin ligase (comprised of Asi1p, Asi2p, and Asi3p) resides in the inner nuclear membrane (Foresti et al., 2014; Khmelinskii et al., 2014). The Asi complex promotes turnover of mislocalized and misfolded soluble and transmembrane proteins (Foresti et al., 2014; Khmelinskii et al., 2014; Natarajan et al., 2020). By virtue of the location of their substrate clientele, Doa10p and the Asi complex are said to mediate inner nuclear membrane-associated degradation (INMAD).

We previously demonstrated that yeast lacking *DOA10* , *ASI1* , and *ASI3* (but not *ASI2* ) exhibit compromised growth in the presence of hygromycin B (Crowder et al., 2015; Niekamp et al., 2019; Runnebohm et al., 2020; Woodruff et al., 2021), an aminoglycoside produced by *Streptomyces hygroscopicus* . Hygromycin B impairs translational fidelity by causing ribosome aminoacyl site distortion, resulting in the production of incorrectly synthesized polypeptides (Brodersen et al., 2000; Ganoza and Kiel, 2001). Sensitivity of a mutant to hygromycin B is consistent with contribution of the mutated gene’s product to protein quality control (PQC ) (Bengtson and Joazeiro, 2010; Crowder et al., 2015; Verma et al., 2013). In this study, we evaluated hygromycin B sensitivity of yeast strains with mutations in genes encoding subunits of two soluble ubiquitin ligases, APC/C ^Cdh1p^ and Slx5p/Slx8p, that also target nuclear substrates.

The anaphase-promoting complex/cyclosome (APC/C) is a multi-subunit soluble ubiquitin ligase that mediates turnover of cell cycle-regulated proteins (e.g. cyclins) (Irniger et al., 1995; Sudakin et al., 1995). APC/C target specificity is determined by the identity of a coactivator subunit, which can be either Cdh1p or Cdc20p (Visintin et al., 1997). APC/C ^Cdh1p^ targets both cytoplasmic and nuclear proteins for degradation and regulates the mitosis-to-G1 transition (Schwab et al., 1997; Visintin et al., 1997). APC/C ^Cdh1p^ was recently shown to promote the turnover of an integral inner nuclear membrane protein in yeast, expanding the panel of ubiquitin ligases that participate in INMAD (Koch et al., 2019). Whether APC mediates PQC in addition to regulatory protein degradation is unknown.

The Slx5p/Slx8p heterodimer was initially characterized as a SUMO (Small Ubiquitin-like MOdifier)-targeted ubiquitin ligase (STUbL), marking for degradation proteins that have first been SUMOylated (Uzunova et al., 2007; Xie et al., 2007). Subsequent studies demonstrated a subset of Slx5p/Slx8p substrates are targeted in a SUMO-independent manner (Xie et al., 2010). Like Doa10p and the Asi complex, Slx5p/Slx8p promotes regulatory protein turnover (e.g. degradation of yeast transcription factor MATα2p (Xie et al., 2010) and SUMO ligase Siz1 (Westerbeck et al., 2014)) and degradation of aberrant proteins (e.g. destruction of a mutated variant of the Mot1p transcription factor (Wang and Prelich, 2009)). The extent to which Slx5p/Slx8p contributes to PQC relative to other nuclear ubiquitin ligases is uncharacterized.

To investigate potential contributions of APC/C ^Cdh1p^ and Slx5p/Slx8p to PQC, we cultured wild type yeast and yeast lacking *DOA10* , *ASI1* , *CDH1* , *SLX5* , or *SLX8* in the absence or presence of increasing concentrations of hygromycin B (Figure 1A). All strains exhibited similar growth in the absence of hygromycin B. As previously observed (Woodruff et al., 2021), *doa10Δ* and *asi1Δ* yeast exhibited marked sensitivity to hygromycin B. Loss of *CDH1* sensitized yeast to hygromycin B to a similar extent as *DOA10* or *ASI1* deletion. *SLX5* and *SLX8* deletion also compromised hygromycin B resistance. *slx8Δ* yeast exhibited modestly greater sensitivity to hygromycin B than *slx5Δ* yeast.

To validate the observation that *CDH1* deletion impairs yeast growth in the presence of hygromycin B, we compared growth of independently generated yeast possessing or lacking *CDH1* (Koch et al., 2019) (Figure 1B). These strains also expressed the inner nuclear membrane APC/C ^Cdh1p^ substrate Mps3p tagged with the V5 epitope. Consistent with data in Figure 1A, *CDH1* deletion diminished resistance to hygromycin B.

To validate the observations made with *slx5Δ* and *slx8Δ* yeast, we analyzed growth of independently generated strains of a distinct genetic background lacking *SLX5* and *SLX8* , individually and in combination (Figure 1C). Indeed, *slx5Δ* yeast, *slx8Δ* yeast, and *slx5Δ* *slx8Δ* yeast each exhibited reduced resistance to hygromycin B. No substantial difference in sensitivity for yeast lacking *SLX5* and *SLX8* was observed, suggesting the differences in fitness of *slx5Δ* and *slx8Δ* yeast observed in our initial experiment (Figure 1A) may reflect genetic background-specific idiosyncrasies.

Our results indicate APC/C ^Cdh1p^ and Slx5p/Slx8p are both critical for optimal fitness in the presence of hygromycin B, which is associated with increased concentrations of aberrant proteins. This is consistent with prominent roles for both ubiquitin ligases in PQC (although our data do not formally exclude non-catalytic function of these proteins in mitigating hygromycin B toxicity). Previous reports established contributions of Slx5p/Slx8p to nuclear PQC (e.g. (Wang and Prelich, 2009)). Further, Slx5p and Slx8p mitigate toxicity associated with polyglutamine-expanded huntingtin protein (Ohkuni et al., 2018).

To our knowledge, a role for APC/C ^Cdh1p^ in PQC has not been suggested. In addition to well-characterized roles in cell cycle-regulated degradation of nuclear and cytoplasmic substrates, our data imply APC/C ^Cdh1p^ may also contribute to destruction of misfolded or otherwise aberrant proteins. Consistent with a role for APC/C ^Cdh1p^ in PQC, negative genetic relationships between *CDH1* and *SLX5* , *SLX8* , and *HRD3* (which encodes a component of the ER PQC HRD ubiquitin ligase) have been detected in large-scale genetic interaction studies (Costanzo et al., 2010; Costanzo et al., 2016; Pan et al., 2006). The nature of aberrancies and subcellular localization of putative APC/C ^Cdh1p^ PQC substrates remains to be determined. In mediating cell cycle progression, Cdh1p recruits APC/C to substrates possessing D-Box and KEN box degradation signals (Burton and Solomon, 2001). Conceivably, translational infidelity induced by hygromycin B could result in the appearance of motifs resembling these degrons in incorrectly synthesized polypeptide molecules; alternatively, APC/C ^Cdh1p^ may exhibit broader substrate specificity than previously appreciated. We note that loss of *CDH1* is synthetically lethal with more than 20 genes (e.g. (Gallegos et al., 2020)). Thus, it is also possible that misfolding and dysfunction of a subset of these gene products (induced by hygromycin B) is lethal in the context of *CDH1* deletion.

Each of the ubiquitin ligases in this study (Doa10p, Asi complex, APC/C ^Cdh1p^ , and Slx5p/Slx8p) promotes regulatory degradation and possesses characterized or implied (based on this study) roles in PQC. To cope with the staggering number of ways protein molecules may conceivably misfold, become damaged, or behave aberrantly, we speculate many ubiquitin ligases with characterized function in regulatory protein turnover (such as APC/C ^Cdh1p^ ) moonlight in PQC.

## Methods

Yeast growth experiments were performed as previously described (Watts et al., 2015). Four μl of sixfold serial dilutions were spotted onto agar plates containing yeast extract-peptone-dextrose medium (Guthrie and Fink, 2004) lacking or possessing hygromycin B (Gibco) at indicated concentrations. Plates were incubated at 30°C and imaged on the indicated days.

## Reagents

**Table:** 

**Name**	**Genotype**	**Figure**	**Reference**
VJY6 (alias MHY500)	*MATa his3-Δ200 leu2-3,112 ura3-52 lys2-801 trp1-1 gal2*	1C	(Chen et al., 1993)
VJY102	*MATa his3Δ1 leu2Δ0 met15Δ0 ura3Δ0 doa10Δ::kanMX4*	1A	(Tong et al., 2001)
VJY360	*MATa his3Δ1 leu2Δ0 met15Δ0 ura3Δ0 asi1Δ::kanMX4*	1A	(Tong et al., 2001)
VJY476 (alias BY4741)	*MATa his3Δ1 leu2Δ0 met15Δ0 ura3Δ0*	1A	(Tong et al., 2001)
VJY643	*MATa his3Δ1 leu2Δ0 met15Δ0 ura3Δ0 slx5Δ::kanMX4*	1A	(Tong et al., 2001)
VJY659	*MATa his3Δ1 leu2Δ0 met15Δ0 ura3Δ0 slx8Δ::kanMX4*	1A	(Tong et al., 2001)
VJY660	*MATa his3Δ1 leu2Δ0 met15Δ0 ura3Δ0 cdh1Δ::kanMX4*	1A	(Tong et al., 2001)
VJY921 (alias HY5850)	*MATa his3Δ1 leu2Δ0 met15Δ0 ura3Δ0 V5-MPS3*	1B	(Koch et al., 2019)
VJY922 (alias HY5901)	*MATa his3Δ1 leu2Δ0 met15Δ0 ura3Δ0 cdh1Δ::kan V5-MPS3*	1B	(Koch et al., 2019)
VJY987 (alias MHY3712)	*MAT* α *his3-Δ200 leu2-3,112 ura3-52 lys2-801 trp1-1 gal2 slx5Δ::kan*	1C	(Xie et al., 2010)
VJY988 (alias MHY3716)	*MAT* α *his3-Δ200 leu2-3,112 ura3-52 lys2-801 trp1-1 gal2 slx8Δ::kan*	1C	(Xie et al., 2010)
VJY989 (alias MHY3861)	*MAT* α *his3-Δ200 leu2-3,112 ura3-52 lys2-801 trp1-1 gal2 slx5Δ::kan slx8Δ::kan*	1C	(Xie et al., 2010)

**Table 1. Yeast strains used in this study.**
